# Testosterone Propionate Exacerbates the Deficits of Nigrostriatal Dopaminergic System and Downregulates Nrf2 Expression in Reserpine-Treated Aged Male Rats

**DOI:** 10.3389/fnagi.2017.00172

**Published:** 2017-05-31

**Authors:** Rui Cui, Yunxiao Kang, Li Wang, Shuangcheng Li, Xiaoming Ji, Wensheng Yan, Guoliang Zhang, Huixian Cui, Geming Shi

**Affiliations:** ^1^Department of Neurobiology, Hebei Medical UniversityShijiazhuang, China; ^2^Department of Human Anatomy, Hebei Medical UniversityShijiazhuang, China

**Keywords:** testosterone propionate, reserpine, nigrostriatal dopaminergic system, oxidative stress, Nrf2-ARE, aged male rats

## Abstract

There is a controversy over the effects of testosterone supplements on dopaminergic function. Both neuroprotective and toxic effects of testosterone supplements are reported. The status of oxidative stress seems to explain the neuroprotective or toxic properties of testosterone. To determine the efficacy of testosterone supplements in different status of oxidative stress, the present studies analyzed the dopamine (DA)-related behaviors and neurochemical indices, as well as markers of nigrostriatal dopaminergic (NSDA) system in reserpine-treated aged male rats followed by testosterone propionate (TP) supplements. The status of oxidative stress of experimental animals was evaluated by analyzing oxidative stress parameters and nuclear factor erythroid 2-related factor 2 (Nrf2)-antioxidant response element (ARE) signaling pathway in substantia nigra (SN). Consistent with our previous studies, TP supplements to 21-month old aged male rats had the beneficial effects on NSDA system and DA-related behaviors and enhanced the antioxidative capabilities in SN. However, the beneficial effects of TP supplements on NSDA system and DA-related behaviors in aged male rats were reversed by reserpine pretreatment to them. Reserpine treatment induced the severe oxidative stress and reduced the expressions of Nrf2, heme oxygenase-1 (HO-1) and NAD(P)H:quinone oxidoreductase-1 (NQO1) in the SN of aged male rats. The TP supplements to reserpine-pretreated aged male rats exacerbated the defects in NSDA system and DA-related behaviors, aggravated oxidative damages and downregulated the expression of Nrf2, HO-1 and NQO1 in the SN. These results suggested that the efficacy of TP supplements on impaired NSDA system was related to the status of oxidative stress in experimental rats.

## Introduction

Oxidative stress is involved in the progression of aging and aging-related dysfunction (Hamilton et al., 2001; Kregel and Zhang, 2007; Wang and Michaelis, 2010), and induces the progressive and irreversible oxidative damages during dopaminergic neurodegeneration (Chen et al., 2008; Hwang, 2013). Regulation of status of oxidative stress in brain might be an effective strategy against the aging-related dopaminergic neurodegeneration (Hwang, 2013; Villar-Cheda et al., 2014). The nuclear factor erythroid 2-related factor 2 (Nrf2), as a transcription factor, controls the basal and inducible expression of an array of antioxidant and detoxification enzymes by binding antioxidant response element (ARE; Carmona-Aparicio et al., 2015; Miller et al., 2015). Activated Nrf2 induces a series of antioxidant genes to protect cells against oxidative stress (Dou et al., 2016). Thus, the activation of Nrf2 is an important approach to attenuate oxidative stress (van Muiswinkel and Kuiperij, 2005; Dou et al., 2016).

Androgens can manipulate the behaviors of organisms (Frye et al., 2008; Zhang et al., 2011) by influencing central dopaminergic activity (Abreu et al., 1988; Kindlundh et al., 2004; de Souza Silva et al., 2009). The supplements of testosterone improve motor symptoms in Parkinson’s disease (Mitchell et al., 2006) and motor behavioral defects in castrated male rats and aged male rats (Zhang et al., 2011; Cui et al., 2012), and enhance dopaminergic activity (Abreu et al., 1988; Zhang et al., 2016). The enhanced dopaminergic activity in aged rats by testosterone seems related to the ameliorated status of oxidative stress (Zhang et al., 2016). Testosterone treatment decreases the vulnerability of neurons of neonatal rats to oxidative stress *in vitro* (Ahlbom et al., 1999) and suppresses orchiectomy-induced oxidative damage and neuronal morphological changes in the hippocampus of adult rats (Meydan et al., 2010). Testosterone supplements to aged male rats activate the Nrf2-ARE pathway and strengthen the antioxidant capability (Zhang et al., 2013, 2016). Androgens exert neuroprotective action against oxidative stress (Ahlbom et al., 1999; Meydan et al., 2010).

Considering the influence of androgens upon the dopaminergic activity and behaviors (Frye et al., 2008; de Souza Silva et al., 2009), as well as the lower testosterone levels in aged men (Hollander et al., 2003), testosterone replacement might be regarded as a therapeutic treatment in aged men against aging-related degenerative disorders (Tenover, 1992; Pike et al., 2009). However, the efficacy of testosterone replacement in aged men is controversial (Okun et al., 2002; Cunningham and Toma, 2011) and both suppressive effects (Dluzen and Ramirez, 1989; Hernandez et al., 1994) and facilitating influences (Abreu et al., 1988; Martínez-Sanchis et al., 2002; de Souza Silva et al., 2009) of testosterone propionate (TP) supplements on the dopaminergic activity are also reported in animal experiments. In addition to the organisms studied (Frye et al., 2010; Borbélyová et al., 2016) and the treatment regimen of androgens (Estrada et al., 2006; Spritzer et al., 2011), a critical factor might determine the efficacy of testosterone supplements. Based on the effects of oxidative stress on aging-related dopaminergic neurodegeneration (Chen et al., 2008; Hwang, 2013), the status of oxidative stress in organisms might be the candidate for the discrepancy when androgens are supplemented.

Reserpine, a blocker of the vesicular monoamine transporter, is a powerful oxidant (Metzger et al., 2002). It increases cytoplasmic dopamine (DA) by preventing the storage of DA in dopaminergic synaptic vesicles. The DA not stored in synaptic vesicles is subjected to degradation either by autoxidation or by oxidative deamination catalyzed by monoamino oxidase (MAO; Lohr, 1991). As a result, the increased cytoplasmic DA causes an acceleration of DA catabolism by MAO as well as an elevated autoxidation of DA, which results in the overproduced reactive species involved in the degenerative processes (Paris et al., 2001; Fuentes et al., 2007) and the alteration in the status of the oxidative stress of dopaminergic nerve terminals (Bilska et al., 2007). Therefore, to investigate the potential influence of oxidative stress on testosterone supplements, the DA-related behaviors and neurochemical indices, as well as markers of nigrostriatal dopaminergic (NSDA) system were analyzed in reserpine-treated aged male rats followed by TP treatment. Meanwhile, oxidative stress parameters and Nrf2-ARE pathway in NSDA system were examined to evaluate the status of oxidative stress of experimental animal models.

## Materials and Methods

### Animals and Housing

Fifty male Wistar rats supplied by the Experimental Animal Center of Hebei Medical University were housed in an air-conditioned room (22 ± 3°C) on a 12 h light–dark cycle (lights on 6:00 AM). Food and water were available *ad libitum*. All of the experimental procedures were conducted in accordance with the rules in the Guidelines for the Care and Use of Mammals in Neuroscience and Behavioral Research and were approved by the Committee of Ethics on Animal Experiments at Hebei Medical University.

### Testosterone Propionate Supplements

All of the rats were randomly divided into the following five groups: 6-month-old group (6Mon, *n* = 10), 22-month-old group (22Mon, *n* = 10), 22-month-old TP group (22Mon-TP, *n* = 10), 22-month-old reserpine group (22MonR, *n* = 10) and 22-month-old reserpine-treated TP group (22MonR-TP, *n* = 10). For 22MonR and 22MonR-TP, the rats at the age of 21 months received a single subcutaneous injection of reserpine (5 mg/kg; CAS: 50-55-5, Tokyo Chemical industry CO., Japan; Bilska et al., 2007). The rats in 22Mon and 22Mon-TP got same treatment with 0.1% glacial acetic acid solution (1 ml/kg) as vehicle instead of reserpine. Forty-eight hours after injection, the rats in 22Mon-TP and 22MonR-TP received daily subcutaneous TP injection (2 mg/kg; Cat. NO.: 35, 805, SERVA Electrophoresis, Germany; Martínez-Sanchis et al., 2002; Chen et al., 2003), which was continued for 4 weeks (28 days). The rats in 22Mon and 22MonR received the same treatment using sesame oil (0.1 ml/kg; MKBH4400V, S3547-250ML, Sigma, USA) as a vehicle.

### Open-Field Test

All of the rats in five groups were used for open-field test. Each rat was placed in an open field chamber (100 × 100 × 40 cm) according to the procedure used in our previous study (Zhang et al., 2011; Cui et al., 2012). The bottom of open field chamber was lined into 25 squares (20 × 20 cm). Every square further consisted of 400 small grills (1 × 1 cm). At the 27th day and 28th day of TP treatment, open-field test was performed for all five groups. Open-field behavior was recorded for 5 min. Three behavioral parameters were observed in the study. They are vertical activity, horizontal activity and total path length, which respectively refer to the total number of erect posture, the total number of square crossings and the total length of crossings of the rat in whole test period. Averaged two measurements at the 27th day and 28th day were made a mean alone to present as an individual result for one rat. The averaged amount of the two measurements of one rat at the 27th day and 28th day was summarized in each group of five groups for later analysis.

### Adhesive Removal Test

Adhesive removal test was performed in home cage as described in our previous study (Cui et al., 2012; Wang et al., 2016) according to Schallert et al. (1982). After adaptive training (5 days, 1 trial per day), all of the rats in each group were tested on the day when last-open-field test ended. Latency to remove stimulus of adhesive paper (Avery) from each side of snout and each forepaw was documented. The first of total three trials was used in data analysis (Schallert et al., 1982).

### Sample Preparation

Following the behavioral tests, the rats in each group were sacrificed by decapitation. The brains were removed quickly. The tissue blocks containing substantia nigra (SN; between 3.00 mm and 4.08 mm rostral to the interaural axis) or caudate-putamen (CPu; between 10.08 mm and 8.64 mm rostral to the interaural axis; Paxinos and Watson, 2007) were dissected on ice-cold plate, using a scalpel for ophthalmic surgery and a stereomicroscopy. The CPu tissue blocks from five rats in each group were processed for analyzing tyrosine hydroxylase (TH) and DA transporter (DAT) at protein level, and those from the other five rats were prepared for detecting DA and its metabolite contents. The unilateral SN tissue blocks from five rats in each group were chosen for analyzing TH and DAT at protein level or used for assaying malondialdehyde (MDA) and reduced glutathione (GSH) contents. The unilateral SN tissue blocks from other five rats in each group were processed for analyzing Nrf2, heme oxygenase-1 (HO-1) and NAD(P)H:quinone oxidoreductase-1 (NQO1) at protein level or for measuring Nrf2, HO-1 and NQO1, as well as TH and DAT at mRNA level. The tissue samples for the protein and mRNA detection were frozen in liquid nitrogen and stored at −80°C for subsequent analysis, and the samples for MDA and GSH as well as DA and its metabolite assay were processed immediately after brain dissection.

### LC–MS/MS Assay

The tissue blocks of CPu were weighed and homogenized in 80% acetonitrile containing 0.1% formic acid (5 μl/mg). The homogenates were centrifuged at 14,000 rpm for 10 min at 4°C. The supernatants were collected and stored at −80°C. DA, 3,4-dihydroxyphenylacetic acid (DOPAC) and homovanillic acid (HVA) in tissue blocks were determined by LC–MS/MS (Sun et al., 2013). The LC separation was performed on Agilent 1200 LC system (Agilent, Santa Clara, CA, USA) using a Synergi Fusion-RP C18 column (50 mm × 3.0 mm, 4 μm) provided by Phenomenex. The MS/MS detection was carried out using a 3200 QTRAP™ LC–MS/MS system (Applied Biosystems, Foster City, CA, USA). The multiple-reaction monitoring mode was used for the quantification.

### Quantitative Real-Time PCR Analysis

Total RNA (2 μg) from SN tissue block was subjected to reverse transcription using random primer to obtain the first-strand cDNA template. Quantitative real-time PCR was performed with 0.8 μl cDNA (diluted 1:10), 2 μl (10 pmoles each) specific primers and 2×GoTaq® Green Master Mix (Promega, Madison, WI, USA) with a final volume of 20 μl. PCR was performed as follows: an initial heat activation step at 95°C for 10 min, followed by 40 cycles with a denaturation step at 95°C for 15 s, an annealing step for 20 s, and an extension step at 72°C for 27 s. The products were analyzed by melting curve to confirm the specificity of amplification. Expression of TH, DAT, Nrf2, HO-1 and NQO1 genes was analyzed using glyceraldehyde-3-phosphate dehydrogenase (GAPDH) as an internal control. The relative quantification was calculated using the 2^−∆∆^^ct^ method. The sets of primers were as follows: TH (5′-GCTTCTCTGACCAGGTGTATCG-3′ and 5′-GCAATCTCTTCCGCTGTGTAT-3′; annealing temperature: 60°C), DAT (5′-ACTCTGTGAGGCATCTGTGTG-3′ and 5′-TGTAACTGGAGAAGGCAATCAG-3′; annealing temperature: 59°C), Nrf2 (5′-GACCTAAAGCACAGCCAACACAT-3′ and 5′-CTCAATCGGCTTGAATGTTTGTC-3′; annealing temperature: 59°C; Wang et al., 2014), HO-1 (5′-TGTCCCAGGATTTGTCCGAG-3′ and 5′-ACTGGGTTCTGCTTGTTTCGCT-3′; annealing temperature: 60°C; Wang et al., 2014), NQO1 (5′-GGGGACATGAACGTCATTCTCT-3′ and 5′-AGTGGTGACTCCTCCCAGACAG-3′; annealing temperature: 62°C; Wang et al., 2014), GAPDH (5′-TGAACGGGAAGCTCACTG-3′ and 5′-GCTTCACCACCTTCTTGATG-3′; annealing temperature: 58°C; Wang et al., 2014). Primer specificity was confirmed by blasting the primer sequence against genomic databases available at NCBI.

### Western Blot Analysis

The tissue blocks for detection of TH and DAT protein levels were homogenized in Radio Immuno Precipitation Assay (RIPA) buffer containing 1% Triton X-100, 0.1% SDS, 0.5% sodium deoxycholate and protease inhibitors (100 μg/ml phenylmethanesulfonyl fluoride, 30 μg/ml aprotinin, 1 mM sodium orthovanadate), and then sonicated for 4 × 10 s. After centrifugation at 12,000× *g* for 20 min at 4°C, the supernatant was collected and centrifuged again as described above. The final resulting supernatant was stored at −80°C until use. Samples from SN (80 μg) or CPu (50 μg) were diluted with 2× sample buffer (50 mM Tris, pH 6.8, 2% SDS, 10% glycerol, 0.1% bromophenol blue, 5% β-mercaptoethanol) and heated for 5 min at 95°C before SDS-PAGE on a 10% gel, and subsequently transferred to PVDF membrane (Millipore). After incubated for 2 h with 5% non-fat dry milk in Tris-buffered saline (TBS) containing 0.05% Tween 20 (TBST; 20 mM Tris-Cl, 137 mM NaCl, 0.1% Tween 20, pH 7.6) at room temperature, the membrane was rinsed in three changes of TBST and then incubated overnight with mouse anti-TH monoclonal antibody (Sigma, T2928, 1:10,000) or rabbit anti-DAT polyclonal antibody (Millipore, Temecula, CA, USA; AB2231, 1:4000) at 4°C. After three washes, the membrane was incubated for 1 h in IRDye® 800-conjugated goat anti-mouse second antibody (1:3000, Rockland) or goat anti-rabbit second antibody (1:3000, Rockland) at room temperature. The membrane was scanned by Odyssey infrared scanner (LI-COR Biosciences). Following stripping, each PVDF membrane was subsequently immunoblotted with mouse anti-β-actin monoclonal antibody (1:6000, Santa Cruz Biotechnology). The labeling densities for TH or DAT were compared with those of β-actin and analyzed by Gel-Pro Analyser Analysis software (Media Cybernetics).

The tissue blocks for detection of Nrf2, HO-1 and NQO1 protein levels were homogenized in an ice-cold lysis buffer (10 mmol/L HEPES, pH 7.9, 10 mmol/L KCl, 0.1 mmol/L EDTA, 1 mmol/L DTT, 0.1 mmol/L EGTA) including protease inhibitors (100 μg/ml phenylmethanesulfonyl fluoride, 30 μg/ml aprotinin, 1 mM sodium orthovanadate) for 15 min. After adding NP-40, the homogenate was centrifuged at 10,000 rpm at 4°C for 3 min, and the supernatant was collected as cytoplasmic protein for HO-1 and NQO1. The pellets were homogenized in an ice-cold lysis buffer (20 mmol/L HEPES, pH 7.9, 400 mmol/L NaCl, 1 mmol/L EDTA, 0.1 mmol/L EGTA) for 15 min. After centrifugation at 12,000 rpm for 10 min at 4°C, the supernatant was collected. Phenylmethanesulfonyl fluoride was added to the supernatant with the final concentration of 1 mmol/L as the nuclear protein for Nrf2. Samples from SN (80 μg) were separated by SDS-PAGE and transferred to PVDF membrane. The membrane was blocked with 5% skimmed milk for 1 h, and then was probed with polyclonal rabbit anti-Nrf2 antibody (Abcam, Cambridge, MA, USA, 1:500), polyclonal rabbit anti-HO-1 antibody (Abcam, Cambridge, MA, USA, 1:200) or polyclonal rabbit anti-NQO1 antibody (Abcam, Cambridge, MA, USA, 1:200) overnight at 4°C. After three washes, IRDye® 800-conjugated goat anti-rabbit second antibody (1:3000, Rockland) incubated with membranes for 1 h at room temperature. The membrane was scanned by Odyssey infrared scanner (LI-COR Biosciences). The densitometry values were normalized with respect to the values of anti-histone 3 (H3, bioWORLD, Dublin, OH, USA, 1:1000) for Nrf2 or anti-β-actin for HO-1 and NQO-1 immunoreactivity. The labeling densities were analyzed by Gel-Pro Analyser Analysis software (Media Cybernetics).

### MDA and GSH Assay

The tissue blocks of SN were homogenized with 10 times (w/v) ice-cold 0.1 M phosphate buffer (PB) at pH 7.4. The homogenates were used to assess reduced GSH and lipid peroxidation product. The levels of GSH and MDA were measured spectrophotometrically using detection kits, following the manufacturer’s instruction (GSH: 016,835; MDA: 022,446, Nanjing Jiancheng Bioengineering Institute, China).

### Serum Testosterone Assay

Samples of trunk blood were collected from the rats used for western blot analysis after decapitation and sat in open microfuge tubes at room temperature for about 30 min to allow the blood to coagulate. Serum samples were prepared by centrifugation and stored at −80°C until assay. Serum testosterone levels were determined by radioimmunoassay using testosterone radioimmunoassay kit (Tianjin Nine Tripods Medical and Bioengineering Co., Ltd. China) according to the protocol of the kit.

### Statistical Analysis

All of the data were described as mean ± SD. The tests of normality (Kolmogorov-Smirnov test) and homogeneity variance (Levene’s test) were applied to all of the data. If both normal distribution (*p* > 0.1) and homogeneity of variance (*p* > 0.1) were found, the parametric test was performed by one-way analysis of variance (one-way ANOVA) followed by a Student-Newman-Keuls (SNK) *post hoc* test for multiple comparisons. Otherwise, nonparametric statistics were done by a Kruskal-Wallis test, where *p* < 0.05, *post hoc* comparisons between groups were performed using the Mann-Whitney *U* test. Differences were considered to be significant when *p* values were less than 0.05. However, for nonparametric statistics using Kruskal-Wallis test (*p* < 0.05), the Mann-Whitney *U* test was done followed by Bonferroni correction. Bonferroni correction was used for multiple comparisons, and the predefined significance level (*p* < 0.05) was reset at *p* < 0.0125 after Bonferroni correction.

## Results

### Serum Testosterone Concentration

The serum testosterone was measured to determine the levels of testosterone in experimental rats. Group differences in the levels of serum testosterone were found among 6Mon, 22Mon, 22Mon-TP, 22MonR and 22MonR-TP rats (*F*_(4,20)_ = 94.247, *p* < 0.01). The levels of serum testosterone were lower in 22Mon rats (1.77 ± 0.70 ng/ml) than that in 6Mon rats (4.59 ± 0.81 ng/ml; *p* < 0.01). Supplements of TP for 4 weeks resulted in higher levels of serum testosterone in 22Mon-TP rats (11.64 ± 1.74 ng/ml) than that in 22Mon rats (*p* < 0.01). No significant difference existed in the levels of serum testosterone between 22Mon and 22MonR rats, as well as between 22Mon-TP and 22MonR-TP rats.

### Behavior Tests

To observe the effects of TP supplements on reserpine-treated aged male rats, DA-related behaviors were analyzed by open-field test and adhesive removal test.

#### Open-Field Behavior

**Figure 1:**
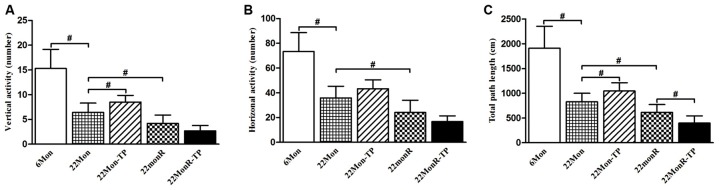
Effects of testosterone propionate (TP) supplements on motor behaviors of reserpine-treated aged male rats. Each rat was individually examined by the open-field test. Open-field behavior (**A**: vertical activity, **B**: horizontal activity, **C**: the total path length) was recorded for 5 min and analyzed *post hoc*. Data were presented as mean ± SD, *n* = 10. ^#^*p* < 0.0125 (Bonferroni correction).

The motor behaviors of experimental rats were observed by detecting vertical activity, horizontal activity and total path length in open-field test. Analysis to the motor behaviors revealed group differences among 6Mon, 22Mon, 22Mon-TP, 22MonR and 22MonR-TP rats in the amount of vertical activity (Figure 1A, χ^2^ = 39.169, *p* < 0.001) and horizontal activity (Figure 1B, χ^2^ = 38.797, *p* < 0.001), as well as total path length (Figure 1C, χ^2^ = 41.604, *p* < 0.001). The *post hoc* test found that the amount of vertical activity and horizontal activity, as well as total path length significantly decreased in 22Mon rats compared with 6Mon rats (*p* < 0.001). The amount of vertical activity (*p* = 0.012) and total path length (*p* = 0.01) significantly increased in 22Mon-TP rats compared with 22Mon rats. Marked reduction in the amount of vertical activity (*p* = 0.011) and horizontal activity (*p* = 0.012), as well as total path length (*p* = 0.01) was detected in 22MonR rats compared with 22Mon rats. Supplements of TP reduced the total path length in reserpine-treated aged male rats (*p* = 0.01).

#### Adhesive Removal Test

**Figure 2:**
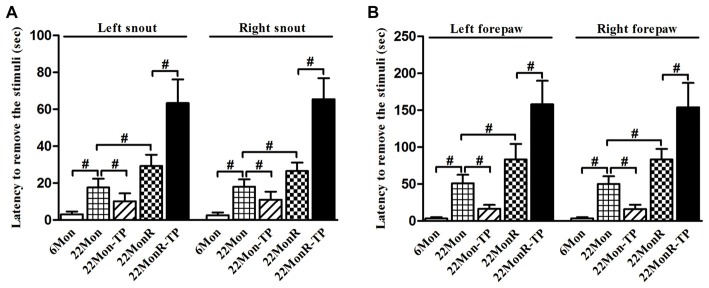
Effects of TP supplements on the adhesive removal test of reserpine-treated aged male rats were examined. Latency to remove stimulus of adhesive paper from each side of snout **(A)** and each forepaw **(B)** was documented. Data were presented as mean ± SD, *n* = 10. ^#^*p* < 0.0125 (Bonferroni correction).

Adhesive removal test demonstrated group differences among 6Mon, 22Mon, 22Mon-TP, 22MonR and 22MonR-TP rats in the latency to remove snout stimuli (Figure 2A, left side of snout: χ^2^ = 45.499, *p* < 0.001; right side of snout: χ^2^ = 45.258, *p* < 0.001) and forepaw stimuli (Figure 2B, left forepaw: χ^2^ = 46.324, *p* < 0.001; right forepaw: χ^2^ = 46.874, *p* < 0.001). The *post hoc* test revealed the longer latency to remove the stimuli from each side of snout and each forepaw in 22Mon rats than in 6Mon rats (*p* < 0.001). In 22Mon-TP rats, the latency to remove the stimuli from each side of snout and each forepaw significantly decreased compared with 22Mon rats (left side of snout: *p* = 0.002; right side of snout: *p* = 0.005; left forepaw: *p* < 0.001; right forepaw: *p* < 0.001). The significant increase in the latency to remove the stimuli from snout and forepaws was observed in 22MonR rats compared with 22Mon rats (left side of snout: *p* = 0.001; right side of snout: *p* = 0.001; left forepaw: *p* = 0.001; right forepaw: *p* < 0.001) and in 22MonR-TP rats compared with 22MonR rats (*p* < 0.001). Supplements of TP increased the latency to remove stimuli in reserpine-treated aged male rats.

### Nigrostriatal Dopaminergic System

The markers of NSDA system were analyzed to investigate the effects of TP supplements on the impaired NSDA system in reserpine-treated aged male rats.

#### DA and its Metabolites

**Figure 3:**
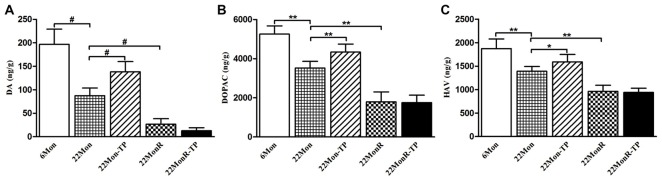
Effects of TP supplements on dopamine (DA) and its metabolites in caudate putamen (CPu) of reserpine-treated aged male rats. DA **(A)**, 3,4-dihydroxyphenylacetic acid (DOPAC; **B**) and homovanillic acid (HVA; **C**) in CPu of rats in each group were detected by LC-MS/MS assay. Data were presented as mean ± SD, *n* = 5. ^#^*p* < 0.0125 (Bonferroni correction); ^*^p < 0.05, ^*^^*^p < 0.01.

Group differences were disclosed among 6Mon, 22Mon, 22Mon-TP, 22MonR and 22MonR-TP rats in DA (Figure 3A, χ^2^ = 22.228, *p* < 0.001), DOPAC (Figure 3B, *F*_(4,20)_ = 69.558, *p* < 0.01) and HVA (Figure 3C, *F*_(4,20)_ = 40.181, *p* < 0.01) content in CPu. *Post hoc* test showed that DA, DOPAC and HVA significantly reduced in 22Mon rats compared with 6Mon rats (DA: *p* = 0.009; DOPAC: *p* < 0.01; HVA: *p* < 0.01), and increased in 22Mon-TP rats compared with 22Mon rats (DA: *p* = 0.009; DOPAC: *p* < 0.01; HVA: *p* < 0.05). The distinguished decrease in DA, DOPAC and HVA was found in 22MonR rats compared with 22Mon rats (DA: *p* = 0.009; DOPAC: *p* < 0.01; HVA: *p* < 0.01). There were no significant differences in the levels of DA, DOPAC and HVA between 22MonR-TP and 22MonR rats.

#### TH and DAT mRNAs

**Figure 4:**
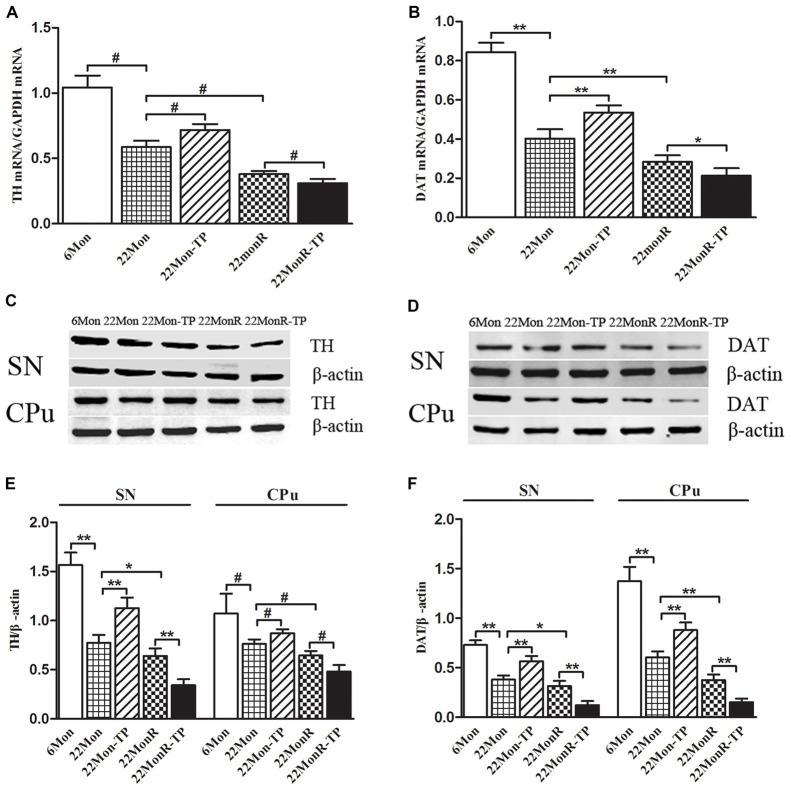
Effects of TP supplements on tyrosine hydroxylase (TH) and DA transporter (DAT) in substantia nigra (SN) and CPu of reserpine-treated aged male rats. TH **(A)** and DAT **(B)** mRNAs were examined by quantitative real-time PCR. TH **(C,E)** and DAT **(D,F)** proteins were detected by Western blot. Data were presented as mean ± SD, *n* = 5. ^#^*p* < 0.0125 (Bonferroni correction); ^*^p < 0.05, ^*^^*^p < 0.01.

Group differences in TH mRNA (Figure 4A, χ^2^ = 22.722, *p* < 0.001) and DAT mRNA (Figure 4B, *F*_(4,20)_ = 179.383, *p* < 0.01) were detected in SN among 6Mon, 22Mon, 22Mon-TP, 22MonR and 22MonR-TP rats. *Post hoc* test found that both TH and DAT mRNAs were lower in 22Mon rats than in 6Mon rats (TH: *p* = 0.009; DAT: *p* < 0.01). Compared with 22Mon rats, TH (*p* = 0.012) and DAT (*p* < 0.01) mRNAs significantly increased in 22Mon-TP rats. The reduced TH and DAT mRNAs were found in 22MonR rats compared with 22Mon rats (TH: *p* = 0.009; DAT: *p* < 0.01) and in 22MonR-TP rats compared with 22MonR rats (TH: *p* = 0.01; DAT: *p* < 0.05). Supplements of TP reduced TH and DAT at mRNA levels in SN of reserpine-treated aged male rats.

#### TH and DAT Proteins

Western blot was used to analyze the levels of TH (Figure 4C) and DAT (Figure 4D) proteins in SN and in CPu. TH and DAT were located at approximately 60 and 80 kDa, respectively. Group differences among 6Mon, 22Mon, 22Mon-TP, 22MonR and 22MonR-TP rats were detected in the levels of TH (Figure 4E, SN: *F*_(4,20)_ = 129.438, *p* < 0.01; CPu: χ^2^ = 21.209, *p* < 0.0001) and DAT (Figure 4F, SN: *F*_(4,20)_ = 122.01, *p* < 0.01; CPu: *F*_(4,20)_ = 163.416, *p* < 0.01) proteins. The *post hoc* test showed the prominent reduction of TH (SN: *p* < 0.01; CPu: *p* = 0.009) and DAT (SN: *p* < 0.01; CPu: *p* < 0.01) in SN and in CPu of 22Mon rats compared with 6Mon rats. Compared with 22Mon rats, the elevated TH (SN: *p* < 0.01; CPu: *p* = 0.009) and DAT (SN: *p* < 0.01; CPu: *p* < 0.01) were observed in 22Mon-TP rats and the decreased TH (SN: *p* < 0.05; CPu: *p* = 0.009) and DAT (SN: *p* < 0.05; CPu: *p* < 0.01) were detected in 22MonR rats. In 22MonR-TP rats, TH (SN: *p* < 0.01; CPu: *p* = 0.009) and DAT (SN: *p* < 0.01; CPu: *p* < 0.01) were lower than in 22MonR rats. Supplements of TP diminished TH and DAT at protein levels in SN and CPu of reserpine-treated aged male rats.

### GSH and MDA

**Figure 5:**
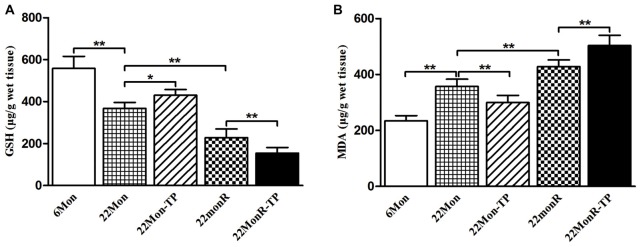
Effects of TP supplements on the levels of oxidative stress in SN of reserpine-treated aged male rats. The levels of glutathione (GSH; **A**) and malondialdehyde (MDA; **B**) were examined spectrophotometrically. Data were presented as mean ± SD, *n* = 5. ^*^p < 0.05, ^*^^*^p < 0.01.

To detect the effects of TP supplements on the levels of oxidative stress in reserpine-treated aged male rats, GSH and MDA, two important parameters of oxidative stress, were measured. Group differences among 6Mon, 22Mon, 22Mon-TP, 22MonR and 22MonR-TP rats were found in the levels of GSH (Figure 5A, *F*_(4,20)_ = 87.785, *p* < 0.01) and MDA (Figure 5B, *F*_(4,20)_ = 78.799, *p* < 0.01) in SN. *Post hoc* test revealed the significant reduction of GSH (*p* < 0.01) and elevation of MDA (*p* < 0.01) in 22Mon rats compared with 6Mon rats. The increased GSH (*p* < 0.05) and decreased MDA (*p* < 0.01) were detected in 22Mon-TP rats and the reduced GSH (*p* < 0.01) and elevated MDA (*p* < 0.01) were observed in 22MonR rats, compared with 22Mon rats. Supplement of TP caused the significant reduction of GSH (*p* < 0.01) and elevation of MDA (*p* < 0.01) in 22MonR-TP rats. Supplements of TP aggravated the oxidative stress in SN of reserpine-treated aged male rats.

### Nrf2-ARE Pathway

To evaluate the effects of TP supplements on Nrf2-ARE pathway in SN of reserpine-treated aged male rats, Nrf2, HO-1 and NQO1 were examined at mRNA and protein levels.

#### Nrf2, HO-1 and NQO1 mRNAs

**Figure 6:**
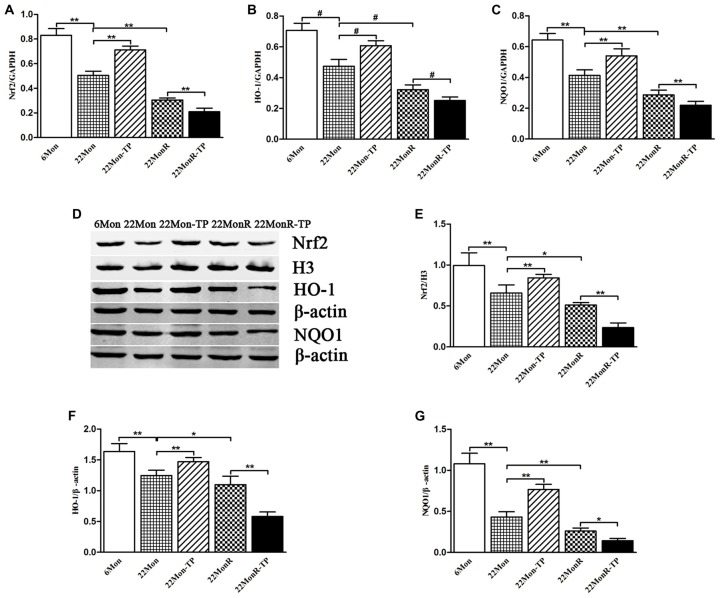
Effects of TP supplements on nuclear factor erythroid 2-related factor 2 (Nrf2), heme oxygenase-1 (HO-1) and NAD(P)H:quinone oxidoreductase-1 (NQO1) in SN of reserpine-treated aged male rats. Nrf2 **(A)**, HO-1 **(B)** and NQO1 **(C)** mRNAs were revealed by quantitative real-time PCR. Nrf2 **(D,E)**, HO-1 **(D,F)** and NQO1 **(D,G)** proteins were detected by Western blot. Data were presented as mean ± SD, *n* = 5. ^#^*p* < 0.0125 (Bonferroni correction); ^*^p < 0.05, ^*^^*^p < 0.01.

Group differences among 6Mon, 22Mon, 22Mon-TP, 22MonR and 22MonR-TP rats were found in Nrf2 mRNA (Figure 6A, *F*_(4,20)_ = 274.248, *p* < 0.01), HO-1 mRNA (Figure 6B, χ^2^ = 22.900, *p* < 0.001) and NQO1 mRNA (Figure 6C, *F*_(4,20)_ = 115.038, *p* < 0.01) in SN. *Post hoc* test showed that Nrf2, HO-1 and NQO1 mRNAs in SN significantly reduced in 22Mon rats compared with 6Mon rats (Nrf2: *p* < 0.01; HO-1: *p* = 0.009; NQO1: *p* < 0.01) and with 22Mon-TP rats (Nrf2: *p* < 0.01; HO-1: *p* = 0.009; NQO1: *p* < 0.01). The marked reduction of Nrf2, HO-1 and NQO1 mRNAs were observed in 22MonR rats compared with 22Mon rats (Nrf2: *p* < 0.01; HO-1: *p* = 0.009; NQO1: *p* < 0.01) and in 22MonR-TP rats compared with 22MonR rats (Nrf2: *p* < 0.01; HO-1: *p* = 0.009; NQO1: *p* < 0.01). Supplements of TP reduced Nrf2, HO-1 and NQO1 at mRNA levels in SN of reserpine-treated aged male rats.

#### Nrf2, HO-1 and NQO1 Proteins

Group differences of the Nrf2 (110 kDa), HO-1 (32 kDa) and NQO1 (31 kDa) proteins in SN (Figure 6D) among 6Mon, 22Mon, 22Mon-TP, 22MonR and 22MonR-TP rats were found (Figure 6E, Nrf2: *F*_(4,20)_ = 54.592, *p* < 0.01; Figure 6F, HO-1: *F*_(4,20)_ = 77.030, *p* < 0.01; Figure 6G, NQO1: *F*_(4,20)_ = 139.64, *p* < 0.01). *Post hoc* test revealed that Nrf2, HO-1 and NQO1 proteins significantly reduced in 22Mon rats compared with 6Mon rats (*p* < 0.01) and with 22Mon-TP rats (*p* < 0.01). Nrf2, HO-1 and NQO1 proteins were found lower in 22MonR rats than in 22Mon rats (Nrf2: *p* < 0.05; HO-1: *p* < 0.05; NQO1: *p* < 0.01) and in 22MonR-TP rats than in 22MonR rats (Nrf2: *p* < 0.01; HO-1: *p* < 0.01; NQO1: *p* < 0.05). Supplements of TP reduced Nrf2, HO-1 and NQO1 at protein levels in SN of reserpine-treated aged male rats.

## Discussion

The present studies showed that TP supplements to aged male rats had beneficial effects on NSDA system and DA-related behaviors. The reduced DA and its metabolites, as well as the decreased TH and DAT in NSDA system of aged male rats were significantly ameliorated by TP supplements. Administration of TP to aged male rats enhanced the expression of Nrf2, HO-1 and NQO1 in SN. However, the beneficial effects of TP supplements on NSDA system and DA-related behaviors in aged male rats were reversed by reserpine pretreatment to them. Reserpine treatment induced the severe oxidative stress and downregulated the expressions Nrf2, HO-1 and NQO1 in the SN of aged male rats. Supplements of TP exacerbated the defects in NSDA system and DA-related behaviors, as well as oxidative damages in reserpine-treated aged male rats. The present results suggested that the efficacy of TP supplements on impaired NSDA system was related to the status of oxidative stress regulated by Nrf2-ARE pathway in experimental rats.

Oxidative stress is one of the important factors implicated in the dopaminergic dysfunctions (Chen et al., 2008; Hwang, 2013). With advancing age, dopaminergic neurons undergo more oxidative stress, as DA auto-oxidation increases markedly with aging (Fornstedt, 1990; Fornstedt et al., 1990; Kumar et al., 2012). Consistent with our previous studies (Cui et al., 2012; Wang et al., 2016; Zhang et al., 2016), the present studies showed the motor deficits, NSDA dysfunctions and oxidative damages in aged male rats, as well as the amelioratory effects of TP administration on them and the involvement of Nrf2-ARE pathway in amelioratory effects of TP supplements in aged male rats (Zhang et al., 2016). However, androgens may not always be neuroprotective (Gavrielides et al., 2006; Cunningham et al., 2009). The different effects of TP supplements to the aged experimental animals were found in the present studies. Supplements of TP ameliorated the behavioral deficits and defects of NSDA system in natural aged male rats, but the same treatments aggravated the deficits in behaviors and in NSDA system in reserpine-treated aged male rats. The status of oxidative stress in them might be critical in determining whether TP plays a neuroprotective or neurotoxic role as suggested by Holmes et al. (2013) *in vitro* studies. Testosterone protects dopaminergic N27 cells from subsequent oxidative insults in a low oxidative stress environment and exacerbates oxidative damages to the cultured cells in high oxidative stress milieus (Holmes et al., 2013). Furthermore, when androgen was used prior to coming oxidative insults, it exerted neuroprotective effects (Cheng et al., 2009; Uchida et al., 2009; Persky et al., 2013; Toro-Urrego et al., 2016). Testosterone protects astrocytic cells against glucose deprivation (Toro-Urrego et al., 2016) and reduces infarction in model of focal ischemia in male rats and male mice (Cheng et al., 2009; Uchida et al., 2009). In the present studies, although the reduced GSH and elevated MDA were observed in 22Mon rats compared with 6Mon rats, which indicated the occurrence of oxidative stress in natural aging rats, much lower GSH and higher MDA was detected in 22MonR rats compared with 22Mon rats.

Reserpine, an oxidative stress inductor, aggravated the oxidative stress in SN of age male rats and reversed the amelioratory effects of TP on the deficits in behaviors and NSDA system of aged male rats. Reserpine alone decreased GSH and increased MDA in the SN of aged male rats. More oxidative damages were induced in the SN of reserpine-treated aged male rats compared with aged male rats. Similar to us, it is found that administration of reserpine to animals dramatically induces the alteration in the status of oxidative stress in CPu (Spina and Cohen, 1989; Bilska et al., 2007), a DA-rich brain structure densely innervated by dopaminergic projection arising from the SN pars compacta. A considerable rise in the levels of oxidized GSH was detected in CPu of reserpine-treated adult rats (Bilska et al., 2007) and mice (Spina and Cohen, 1989). Pretreatment of androgens promotes the expression of different components of the antioxidant defense systems (Ahlbom et al., 1999; Toro-Urrego et al., 2016) to resist the coming oxidative injuries. Under the physiology condition, Nrf2 is sequestered in the cytoplasm by the repressor protein Keap1. However, under conditions of stress, Nrf2 is released from Keap1 and translocates to the nucleus (Carmona-Aparicio et al., 2015). Subsequently, Nrf2 binds to ARE to activate the transcription of corresponding downstream antioxidant genes, such as HO-1 and NQO1 (Prestera et al., 1995; Lee and Johnson, 2004; Dou et al., 2016) to suppress the oxidative stress and maintain the redox balance. In the present studies, we found TP supplements to natural aged male rats at 21-month old upregulated the expression of Nrf2, HO-1 and NQO1 in the SN. Activation of Nrf2-ARE pathway protects neurons against oxidative and excitotoxic damages (Ahmad et al., 2006; Chen et al., 2011; Wang et al., 2014; Carmona-Aparicio et al., 2015). Disruption of Nrf2-ARE pathway results in an increased susceptibility to oxidative insults and other toxicants (Kwak et al., 2002). Significant reduction in the expressions of Nrf2, HO-1 and NQO1 was detected in reserpine-treated aged male rats, which indicated the aggravated antioxidative capability. Thus, the exacerbation effects of TP supplements on the deficits of NSDA system in reserpine-treated aged male rats might be related to the increased oxidative stress due to reserpine-downregulating Nrf2-ARE pathway in some extent.

In the present studies, only reserpine was chosen as oxidative stress inductor. More methodology for inducing oxidative stress should be employed to testify the potential influence of oxidative stress on testosterone supplements in the following studies. Furthermore, contrary to our finding, reserpine upregulates Nrf2 epigenetically in mouse skin epidermal JB6 P+ cells (Hong et al., 2016). The following reason might explain the response differences to reserpine. It is found that Nrf2 differs between various neuronal subpopulations and regulates different gene products, such as in the hippocampal neurons vs. nigral neurons (Kraft et al., 2004). The similar situation might occur in nigral neurons vs. mouse skin epidermal JB6 P+ cells. Whether it is true or not is necessary to be investigated.

In conclusion, TP supplements enhanced the activity of NSDA system and ameliorated the motor deficits of aged male rats. Reserpine pretreatment induced the elevated oxidative stress, downregulated Nrf2 expressions and reversed the amelioratory effects of TP supplements on the deficits in NSDA system of aged male rats. The status of oxidative stress might determine the efficacy of testosterone supplements to aged male rats.

## Author Contributions

RC and YK performed PCR, western blot experiments, analyzed the data and wrote the manuscript. LW and SL performed the biochemical experiments and LC–MS/MS assay and contributed to drafting. XJ, WY and GZ carried out behavioral experiments and the measurement of serum testosterone levels. HC interpreted data and revised the manuscript. GS designed the study, analyzed the data and revised the manuscript. All authors have read and approved the final version of the manuscript.

## Conflict of Interest Statement

The authors declare that the research was conducted in the absence of any commercial or financial relationships that could be construed as a potential conflict of interest.

## Abbreviations

ARE: antioxidant response element
CPu: caudate putamen
DA: dopamine
DAT: dopamine transporter
DOPAC: 3,4-dihydroxyphenylacetic acid
GSH: glutathione
HO-1: heme oxygenase-1
HVA: homovanillic acid
MDA: malondialdehyde
NQO1: NAD(P)H:quinone oxidoreductase-1
Nrf2: nuclear factor erythroid 2-related factor 2
NSDA: nigrostriatal dopaminergic
SN: substantia nigra
TH: tyrosine hydroxylase
TP: testosterone propionate.
